# Cross-validation of independent ultra-low-frequency magnetic recording systems for active fault studies

**DOI:** 10.1186/s40623-018-0823-7

**Published:** 2018-04-18

**Authors:** Can Wang, Chen Bin, Lilianna E. Christman, Jonathan M. G. Glen, Simon L. Klemperer, Darcy K. McPhee, Karl N. Kappler, Tom E. Bleier, J. Clark Dunson

**Affiliations:** 1 U.S. Geological Survey, Menlo Park, CA 94025 USA; 2 Institute of Geophysics, China Earthquake Administration, Beijing, 100081 People’s Republic of China; 3 Department of Geophysics, Stanford University, Stanford, CA 94305-2215 USA; 4 QuakeFinder Inc., Palo Alto, CA 94306 USA; 5 Myriad Botanical Gardens, Oklahoma City, OK 73102 USA; 6 U.S. Geological Survey, Reston, VA 20192 USA

**Keywords:** Ultra-low frequency (ULF), Magnetic observatory, Time-series analysis, Magnetic field, Instrumental noise

## Abstract

When working with ultra-low-frequency (ULF) magnetic datasets, as with most geophysical time-series data, it is important to be able to distinguish between cultural signals, internal instrument noise, and natural external signals with their induced telluric fields. This distinction is commonly attempted using simultaneously recorded data from a spatially remote reference site. Here, instead, we compared data recorded by two systems with different instrumental characteristics at the same location over the same time period. We collocated two independent ULF magnetic systems, one from the QuakeFinder network and the other from the United States Geological Survey (USGS)-Stanford network, in order to cross-compare their data, characterize data reproducibility, and characterize signal origin. In addition, we used simultaneous measurements at a remote geomagnetic observatory to distinguish global atmospheric signals from local cultural signals. We demonstrated that the QuakeFinder and USGS-Stanford systems have excellent coherence, despite their different sensors and digitizers. Rare instances of isolated signals recorded by only one system or only one sensor indicate that caution is needed when attributing specific recorded signal features to specific origins.
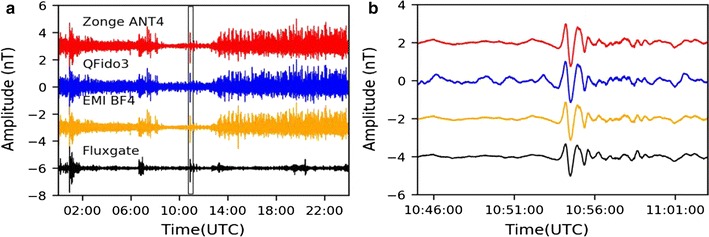

## Introduction

There have been numerous reports of anomalous ultra-low-frequency (ULF, 0.01–10 Hz) signals in magnetic data preceding earthquakes (e.g., Bleier et al. 2009; Dunson et al. 2011; Fraser-Smith et al. 1990; Hayakawa et al. 1996; Kopytenko et al. 1993; Varotsos et al. 1999), and it has been argued that these signals are precursors genetically related to impending earthquakes and arising within the earth (“tectonic signals”). However, an almost equal number of studies have attempted to show that these undoubtedly unusual “signals” are not genetic precursors to earthquakes (e.g., Campbell 2009; Thomas et al. 2009a, b; Tzanis et al. 2000). Some of these “refuting” studies have suggested that putative earthquake precursors may represent sensor-system malfunctions (“amplifier instrumental noise”) (e.g., Thomas et al. 2009a). Other refuting studies implicate natural variability of the global magnetic field (e.g., Campbell 2009; Thomas et al. 2009b) that is largely due to solar-terrestrial interaction (hereinafter “geomagnetic signals”). Yet, other refuting studies assert that claimed electromagnetic (EM) precursors confuse anthropogenic signals (or “cultural noise”) with fields generated internally in the earth’s crust (e.g., Tzanis et al. 2000). However, a shortfall of almost all previous reports (whether claiming or refuting precursory magnetic activity) is that they describe signals recorded at a single station that lack external corroboration (e.g., Fraser-Smith et al. 1990; Geller 1996; Hayakawa et al. 1996; Kopytenko et al. 1993; Kappler et al. 2010; Varotsos et al. 1999). Thus, it is important to characterize ULF-EM data as thoroughly as possible in order to be able to distinguish between the different signals (geomagnetic and tectonic) and noise sources [instrumental, cultural, and other local (e.g., environmental)]. As ULF magnetic stations are not widespread, comparison between networks increases the potential to characterize the spatial variation of anomalous signals and assess the existence of ULF signals of tectonic origin. A thorough characterization of each system and comparison of the signals measured by each system are needed before multiple systems can be used to jointly assess the nature and origins of signals.

The characteristics of geomagnetic signals considered to be potential earthquake precursors are highly variable between, and even within, peer-reviewed studies. Fraser-Smith et al. (1990) and Bernardi et al. (1991) described two different ULF phenomena prior to the Ms = 7.1 Loma Prieta, CA, earthquake: a narrowband (10^−6^ octave) ≤ 30 dB amplification of magnetic fluctuations at 0.1 Hz on a horizontal magnetometer lasting 3 weeks; and a wideband (three octave, 0.01–10 Hz) amplification with peak ~ 40 dB no more than 30 min in duration. In contrast to these measurements on a single horizontal magnetometer, Hayakawa et al. (1996) showed a month-long increase within the 0.01–0.05 Hz band in the ratio of vertical to horizontal amplitudes before a Ms = 7.1 Guam earthquake, whereas Varotsos et al. (1999, 2006) described > 1 s magnetic pulses occurring on all three components of a magnetometer during 2 days over 3 weeks prior to a Ms = 7.1 earthquake in Greece, although they relied more heavily on signals of ~ 10^−5^ V/m recorded for minutes to hours on electric dipoles. Most recently, the QuakeFinder network of magnetometers (see below) has been used to catalo and categorize thousands of transient magnetic pulses of duration 1–30 s, some prior to moderate earthquakes (Bleier et al. 2009; Dunson et al. 2011) and others apparently unassociated with earthquake activity (Kappler et al. 2017). Given the broad range of possible electromagnetic signatures available to study, some magnetic observatories deliberately work to remove pulses that are likely related to cultural noise (Minamoto et al. 2011; Nagamachi et al. 2013). By contrast, we have chosen to focus on transient pulses, not only as these have been recently and extensively studied (Bleier et al. 2009; Dunson et al. 2011; Kappler et al. 2017), but also as they are crucial to some of the most controversial claims of ULF electromagnetic precursors (e.g., Varotsos et al. 1999, 2006 and references cited therein).

**Fig. 1:**
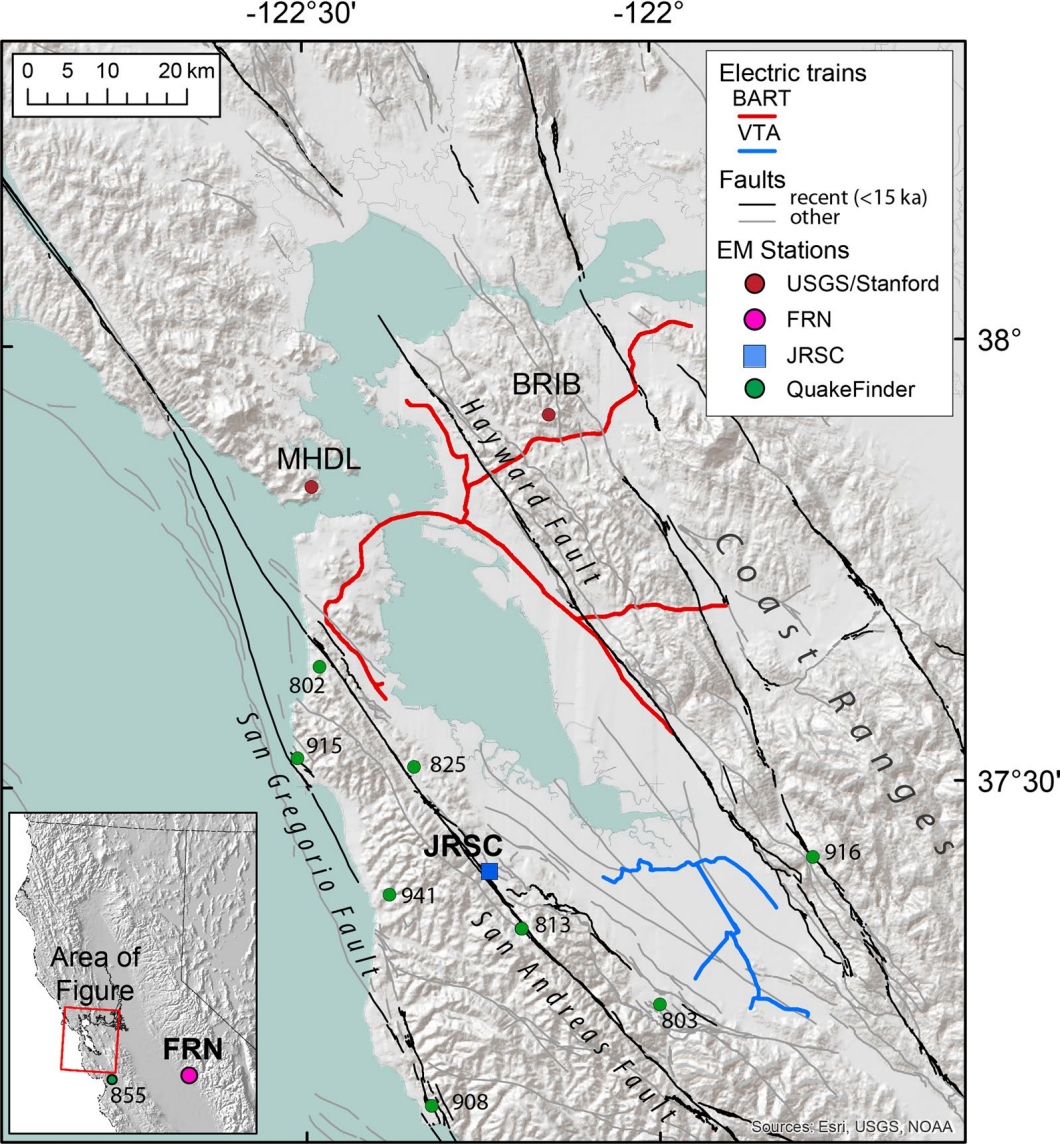
Location map of the greater Bay Area, California (CA) with hill-shaded topography. Blue square: USGS-Stanford permanent electromagnetic observatory and QuakeFinder temporary magnetic systems at Jasper Ridge Biological Preserve (JRSC); pink circle: USGS Fresno (FRN) magnetic station (in inset); green circles: QuakeFinder permanent EM stations in San Francisco Bay Area; red circles: other USGS-Stanford EM stations MHDL and BRIB. Gray and black lines: faults. Red and blue lines: electric trains, respectively, BART tracks and VTA light rail

This report examines 6 weeks of data from two different and independent ULF magnetic recording systems temporarily collocated at Stanford University’s Jasper Ridge Biological Preserve (JRSC) (Fig. 1). One is the Jasper Ridge permanent station (JRSC) managed by the United States Geological Survey (USGS), Stanford University, and the Berkeley Seismological Laboratory (hereafter “USGS-Stanford network”; Bijoor et al. 2005; Karakelian et al. 2000), whereas the other was temporarily deployed by QuakeFinder (Cutler et al. 2008). By collocating the two systems, we were able to characterize their system responses to a wide range of signals and noises, thereby enabling future inter-station comparisons between different types of sensors located at different sites. Both datasets were further compared to a remote reference station maintained by the USGS National Geomagnetism Program at Fresno, CA (FRN) (Fig. 1) in order to distinguish external signals from local and cultural noise. Although it would also be possible to compare our collocated data with that of other permanent QuakeFinder and USGS-Stanford stations in the Bay Area to further address the local versus regional nature of signals (Fig. 1), in this work we focus on system responses of collocated sensors, in order to achieve a more quantitative comparison of different sensor systems.

## Data acquisition

**Fig. 2:**
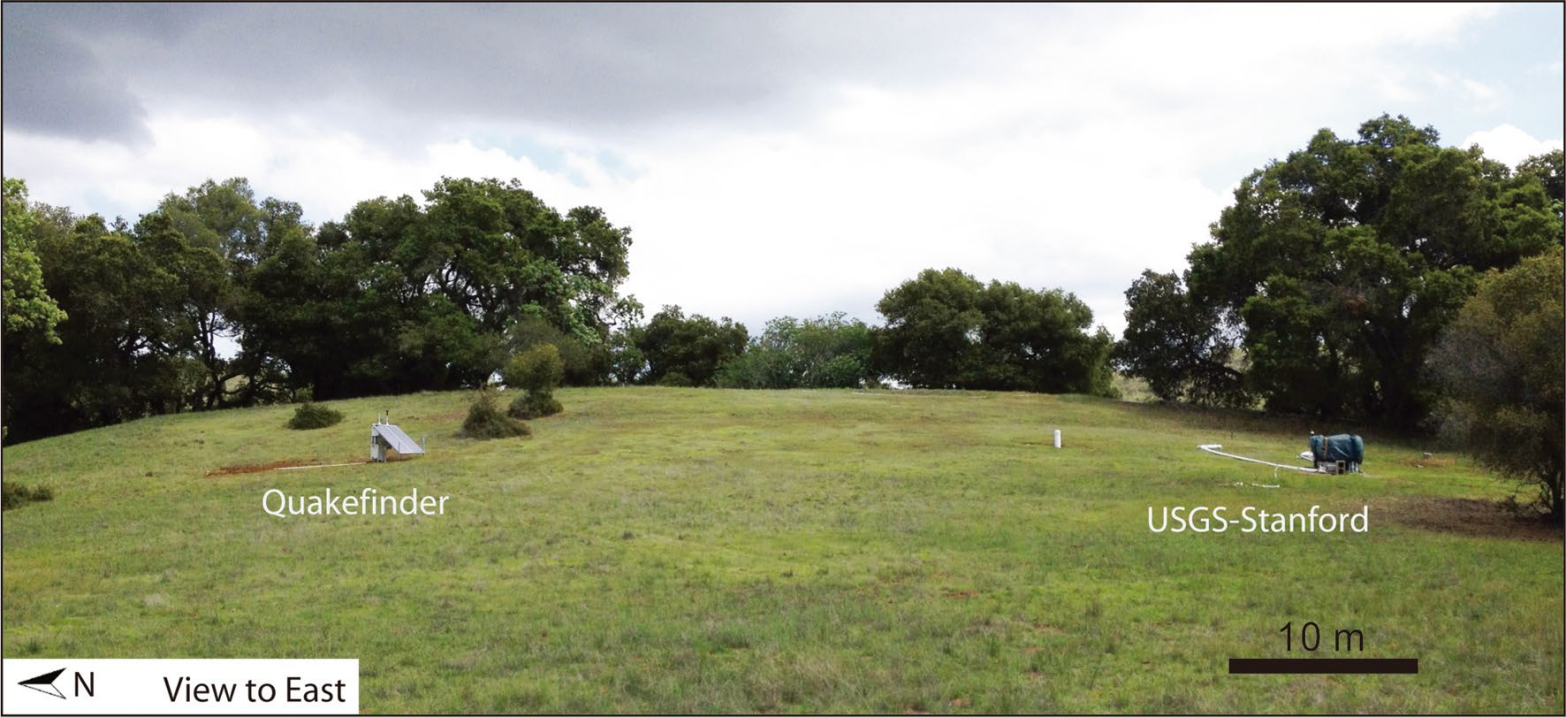
Photograph of QuakeFinder temporary installation (left, north) and USGS-Stanford permanent electromagnetic observatory JRSC (right, south)

The USGS-Stanford electromagnetic network includes JRSC, a permanent site at the Jasper Ridge Biological Preserve of Stanford University. From March 31, 2014 to May 13, 2014, QuakeFinder maintained a temporary station adjacent to JRSC, using equipment standard to their own network operations (Fig. 2). The two systems, consisting of magnetic induction coils, were separated by ~ 50 m to eliminate interference, but both systems shared the low-noise local environment (~ 100 m from the nearest dirt road and parking area; ~ 50 m from the closest 110 V power line, which serves only the collocated geophysical experiments). Each coil was buried ~ 30 cm below ground surface and at least 1 m away from the other coils. Of course, these sites are located within the San Francisco Bay Area and are thus continuously affected by significant anthropogenic sources, including the region-wide Bay Area Rapid Transit (BART) DC electric railway line, the Valley Transportation Authority (VTA) light rail (Fig. 1), and other additional local interferences such as the SLAC Linear Accelerator Center (SLAC) also housed on Stanford campus lands. BART and VTA, which operate on 1000 and 525–875 V direct-current systems, respectively (Fraser-Smith and Coates 1978; Liu and Fraser-Smith 1996), are major sources of ULF magnetic noise in the Bay Area and surrounding regions, except for 2 h in the early morning (02:00–04:00 local time) when the electric trains are not usually in operation.

## System configurations and response functions

**Fig. 3:**
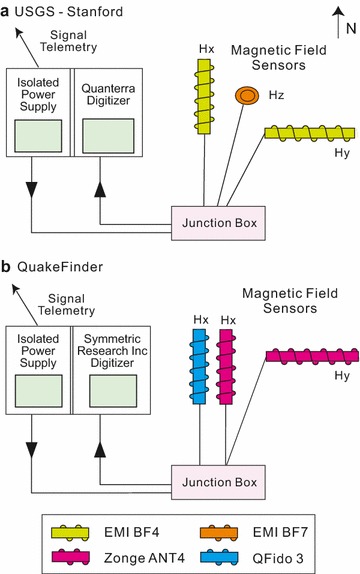
Schematic equipment diagrams, as installed at JRSC: **a** USGS-Stanford and **b** QuakeFinder. *H*_*x*_, *H*_*y*_, and *H*_*z*_ represent magnetic coils oriented in the magnetic north, magnetic east, and vertical directions, respectively. Note that the USGS-Stanford electric dipoles are not shown

The USGS-Stanford system consists of three Electromagnetic Instruments Inc. (EMI) sensors, oriented with their coil axes aligned in the geomagnetic north–south (016°, *H*_*x*_), east–west (106°, *H*_*y*_), and vertical (*H*_*z*_) directions (Fig. 3a). The horizontal sensors are the BF4 model (https://www.slb.com/≃/media/Files/rd/technology/product_sheets/emi_bf_4.pdf), and the vertical sensor is the very similar BF7 (http://www.slb.com/~/media/Files/rd/technology/product_sheets/emi_bf_7_sensor.pdf). The magnetic data were digitally recorded at 40 Hz, on a 24-bit Quanterra Q330 digitizer (http://www.q330.com/), and telemetered to the Berkeley Northern California Earthquake Data Center (www.quake.geo.berkeley.edu). QuakeFinder deployed two modified Zonge ANT-4 sensors (http://zonge.com/instruments-home/instruments/geophysical-sensors-magnetometers/) and one QFido3 sensor (developed in-house by QuakeFinder) that has a lower preamp gain (Fig. 3b). No vertical sensor was deployed, to avoid excessive ground disturbance for the short-term experiment. One ANT-4 sensor and one QFido3 sensor were aligned in the geomagnetic north–south direction, and the other ANT-4 sensor was aligned geomagnetic east–west. All three QuakeFinder magnetometers were recorded at 50 Hz, digitized using a Symmetric Research Inc. digitizer with eight independent channels and a 24-bit digitizer (http://www.symres.com/), and telemetered to the QuakeFinder offices (www.quakefinder.com) in Palo Alto, CA. Both the ANT4 and QFido3 sensors have been outfitted with 3-pole high-cut filters to help attenuate powerline noise, and an additional 5-pole high-cut filter nominally at 12 Hz is applied by an analog board.

**Fig. 4:**
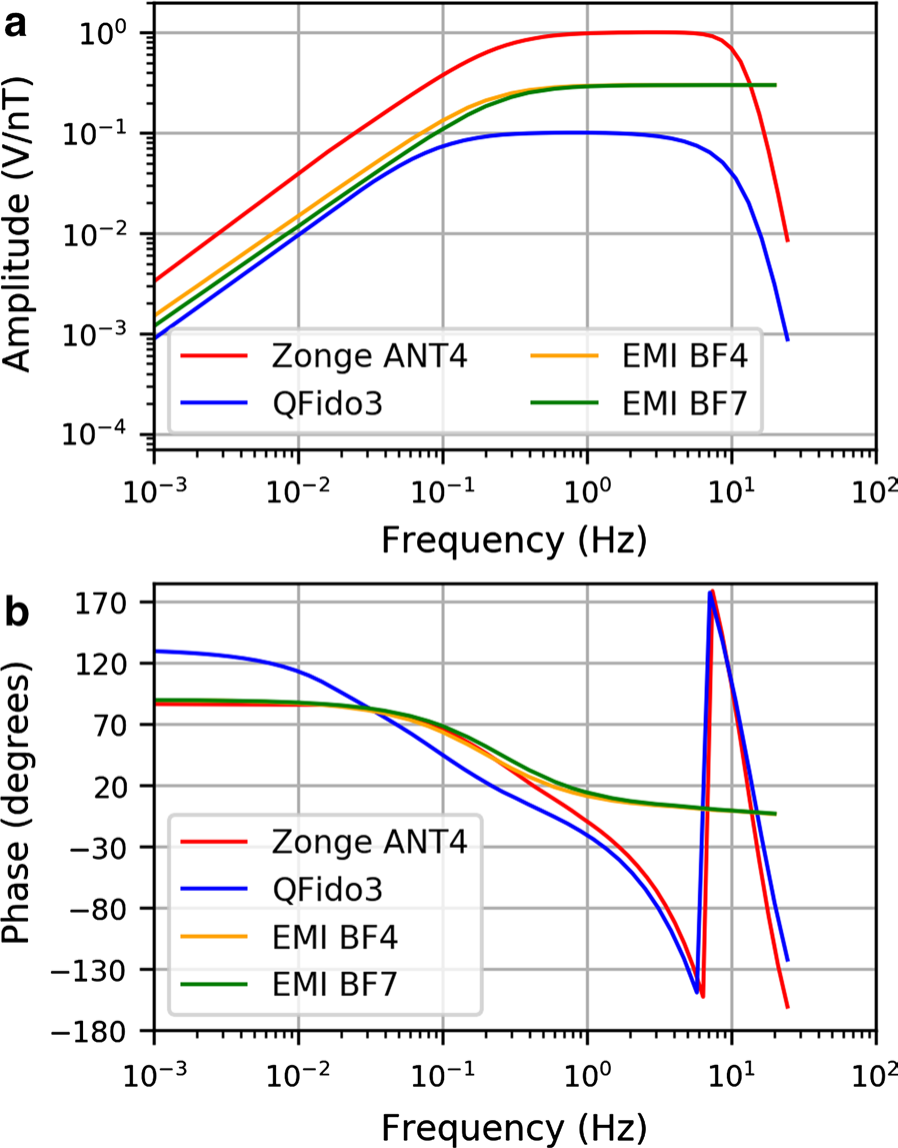
**a** Amplitude spectra and **b** phase spectra of the response function of EMI BF4, EMI BF7, Zonge ANT4, and QFido3 coils. Phase shifts are plotted between − 180° and 180°. The QuakeFinder system low-pass filters manifest as “phase wrapping” near 10 Hz in **b**

The voltage output of the instruments is a complex-valued linear function of the ambient magnetic field. The amplitude and phase spectra of the instrument response functions of the three coil types (Fig. 4) show the order-of-magnitude range of sensitivity (*V*/*nT*) of the three instruments in the bandwidth of greatest interest (100 s to 10 Hz), as well as phase shifts inherent in the transducers. The response of the analog board was measured by QuakeFinder using a HP3562A Signal Analyzer, and incorporated into the spectra shown in Fig. 4. The effect of the low-pass filters on the QuakeFinder system can be seen clearly on the right-hand side of Fig. 4a, b. All transfer functions used for the ANT4, QFido3, BF4, and BF7 (vertical coil at JRSC) models were representative manufacturer’s calibration curves for these models. FRN utilizes a Narod fluxgate magnetometer (Narod and Bennest 1990) sampled at 1 Hz (https://geomag.usgs.gov/monitoring/observatories/fresno/).

## Data processing

**Fig. 5:**
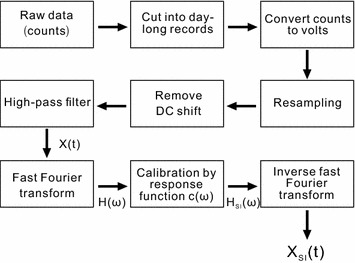
Schematic data-processing sequence. The stages of the transformation to nT from *X*(*t*) to *X*_SI_(*t*) are inserted between the boxes representing operations in the processing flow

All data are recorded in units of digital counts (Fig. 5). We first cut data into day-long records and normalized all data by their nominal counts-per-volt (cpv) conversion factors—nominally 4.2 × 10^5^ and 4.0 × 10^5^ for the Quanterra (USGS-Stanford) and Symmetric (QuakeFinder) digitizers, respectively. To simplify comparative plotting and processing, the 40 Hz data from the USGS-Stanford array (EMI, BF4, and BF7) were resampled to 50 Hz. Uniformly sampled time series in units of volts were then processed on a day-by-day basis to generate time series in nT. A third-order Butterworth zero-phase high-pass filter with a low-cut frequency of 1000 s was used to eliminate longer-period signals.

Data calibration was applied in the frequency domain, using an arbitrary but fixed recorded voltage time series *X*(*t*), which becomes the spectral series *H*(*ω*) under fast Fourier transform. These data were associated with an instrument whose complex response function *c*(*ω*) is shown in Fig. 4. Spectra in SI units (*H*_SI_) were approximated by point-by-point division *H*(*ω*)/*c*(*ω*). The calibrated time series was the real part of the inverse fast Fourier transform of *H*_SI_ (Fig. 5).

## Data description

### Data characterized in frequency domain

**Fig. 6:**
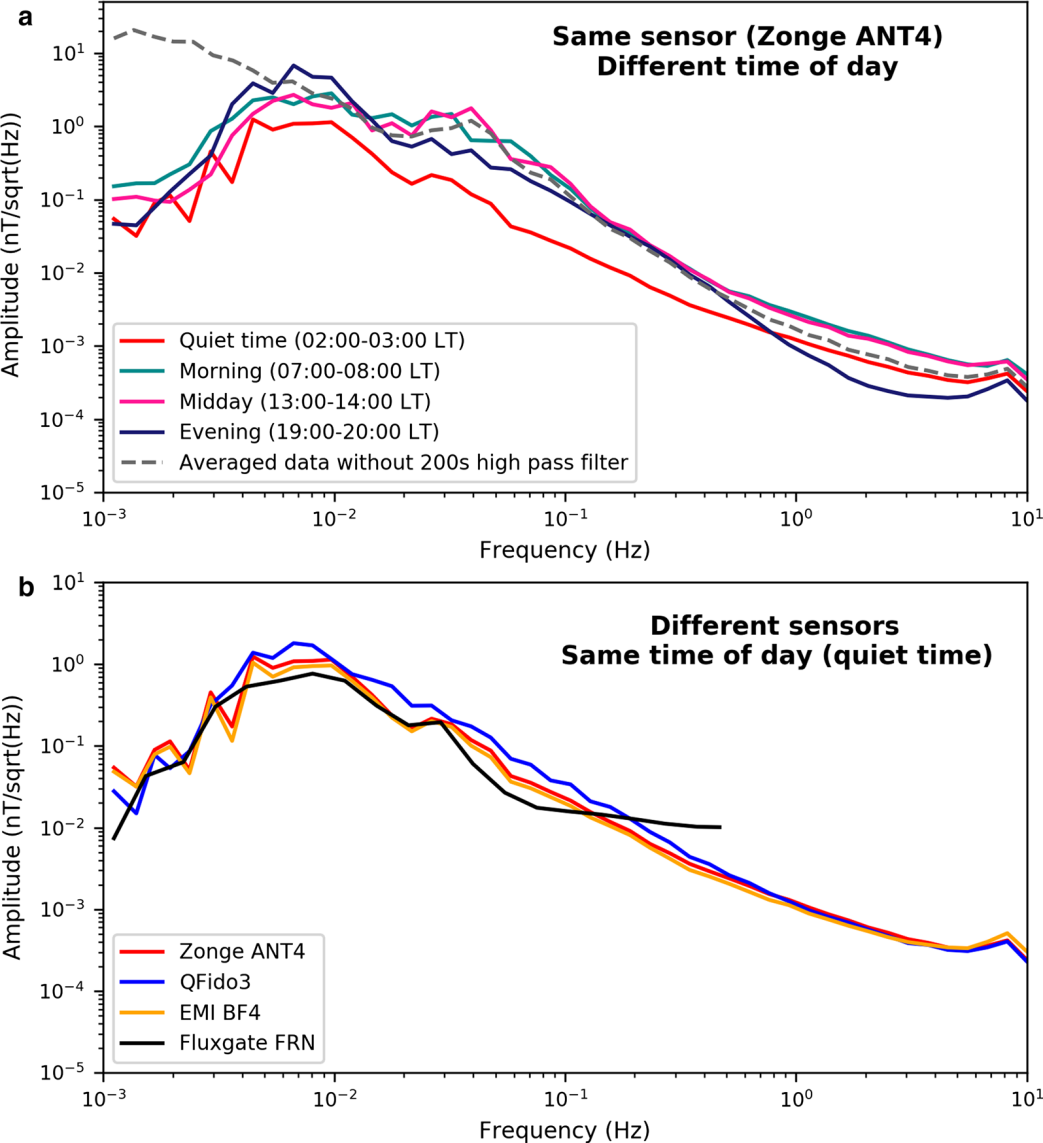
Comparisons of amplitude spectra between different times of day and between different instruments. **a** Zonge ANT4 north-oriented coil averaged over 1 h at four different times during April 19, 2014. Solid lines show data filtered from 200 s to 10 Hz. Dashed line is the average spectrum over the whole day, without a low-pass filter (but still with 10 Hz anti-alias filter). **b** Amplitude spectra of Zonge ANT4, QFido3, and EMI BF4 coil magnetometers during the “quiet period” (02:00–03:00 local time) on April 19, 2014, also compared with the fluxgate magnetometer at the Fresno (FRN) observatory

Figure 6a shows typical band-averaged amplitude spectra of background geomagnetic activity recorded on the QuakeFinder ANT4 north–south sensor at various times throughout the day. The background signals we measured were largely “external signals,” originating from natural sources in the magnetosphere and ionosphere. At our highest recorded frequencies, the first Schumann resonance (Nickolaenko and Hayakawa 2014) produced an increase in geomagnetic activity in the range 5–10 Hz. At lower frequencies, there was a monotonic increase in activity with decreasing frequency. Amplitude spectra clearly showed higher power during the day and the lowest power at night, during the “quiet time” (02:00–03:00 local time) when the electric trains were inactive. At 0.03–0.05 Hz, around which the electric-train signals are centered, the background noise was 10 times greater during the day than during the quiet time (Karakelian et al. 2000).

The amplitude spectra also showed good coherence between different sensor models (Fig. 6b). The first Schumann resonance peak (8 Hz) had an amplitude of around 0.45 pT/sqrt(Hz) as recorded by all three north–south sensors and consistent with the expected values (e.g., Heckman et al. 1998). Over most of the frequency band of interest, the QFido3 was, as expected, the noisiest system.

**Fig. 7:**
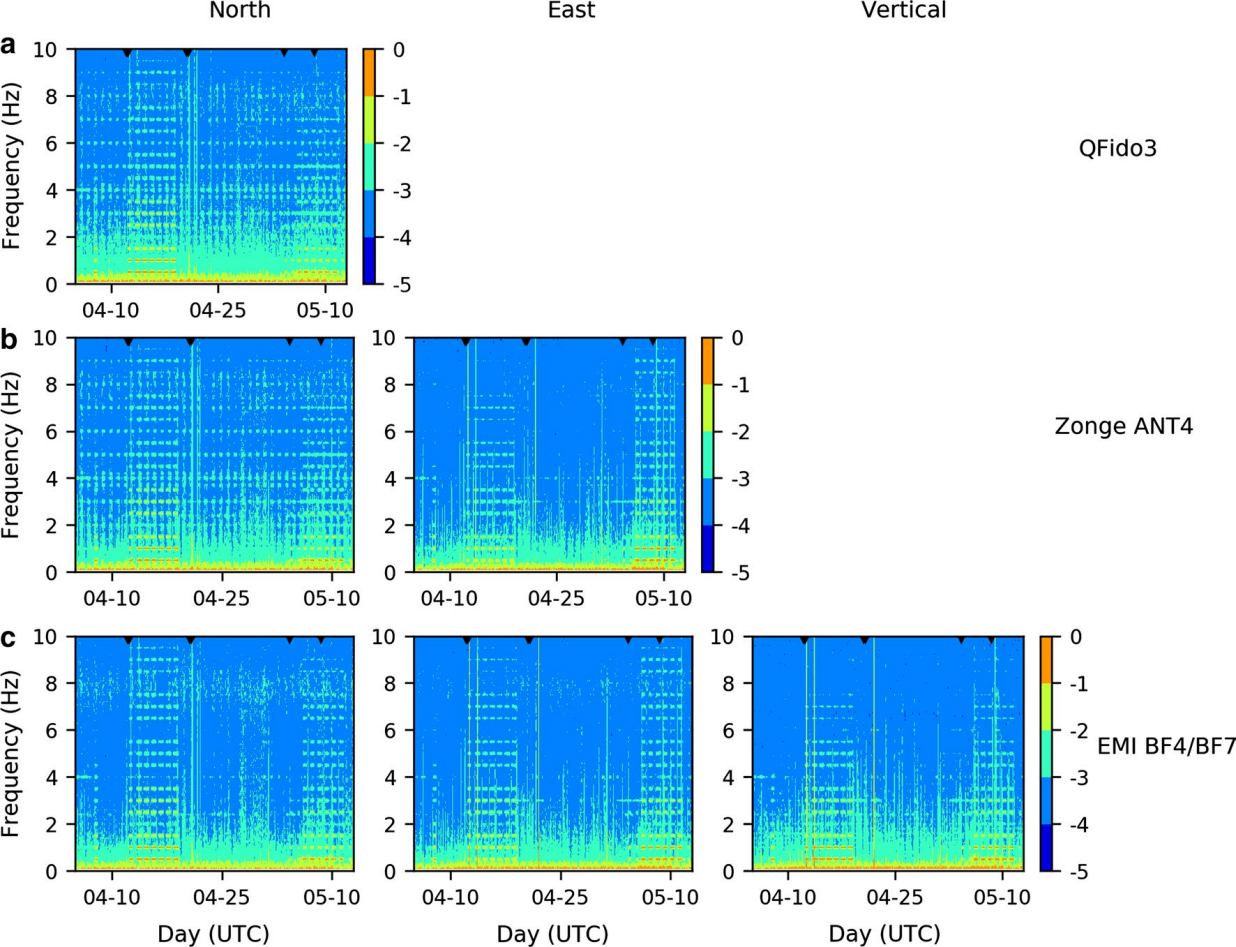
Spectrograms for the time interval April 5, 2014 to May 12, 2014. **a** QFido3 north coil; **b** Zonge ANT4 north and east coils; **c** EMI BF4 north, east, and EMI BF7 vertical coils. The black triangles along the upper plot border show times of high geomagnetic activity (Ap > 29)

Spectrograms of the different coils also showed good correlation (Fig. 7). Several broadband signals appeared as anomalously large amplitudes over short time periods; these generally occurred on days of anomalously high global geomagnetic activity (geomagnetic storms are marked as black triangles in Fig. 7). An interesting phenomenon, which was present in all datasets but was not obvious from the time series or spectra, was a banded signature with harmonics at multiples of 0.5 Hz, occurring at the same time each day during April 12–18 and May 5–10. These signals were probably caused by the SLAC National Accelerator Laboratory, which has its injector ~ 1 km distant from our magnetometers. During the period in question, SLAC was using parts of the 3 km linear accelerator comprising 65 MW pulsed klystrons driving accelerator structures with beam rates at, variably, 1, 10, and 120 Hz.

### Data characterized in time domain

**Fig. 8:**
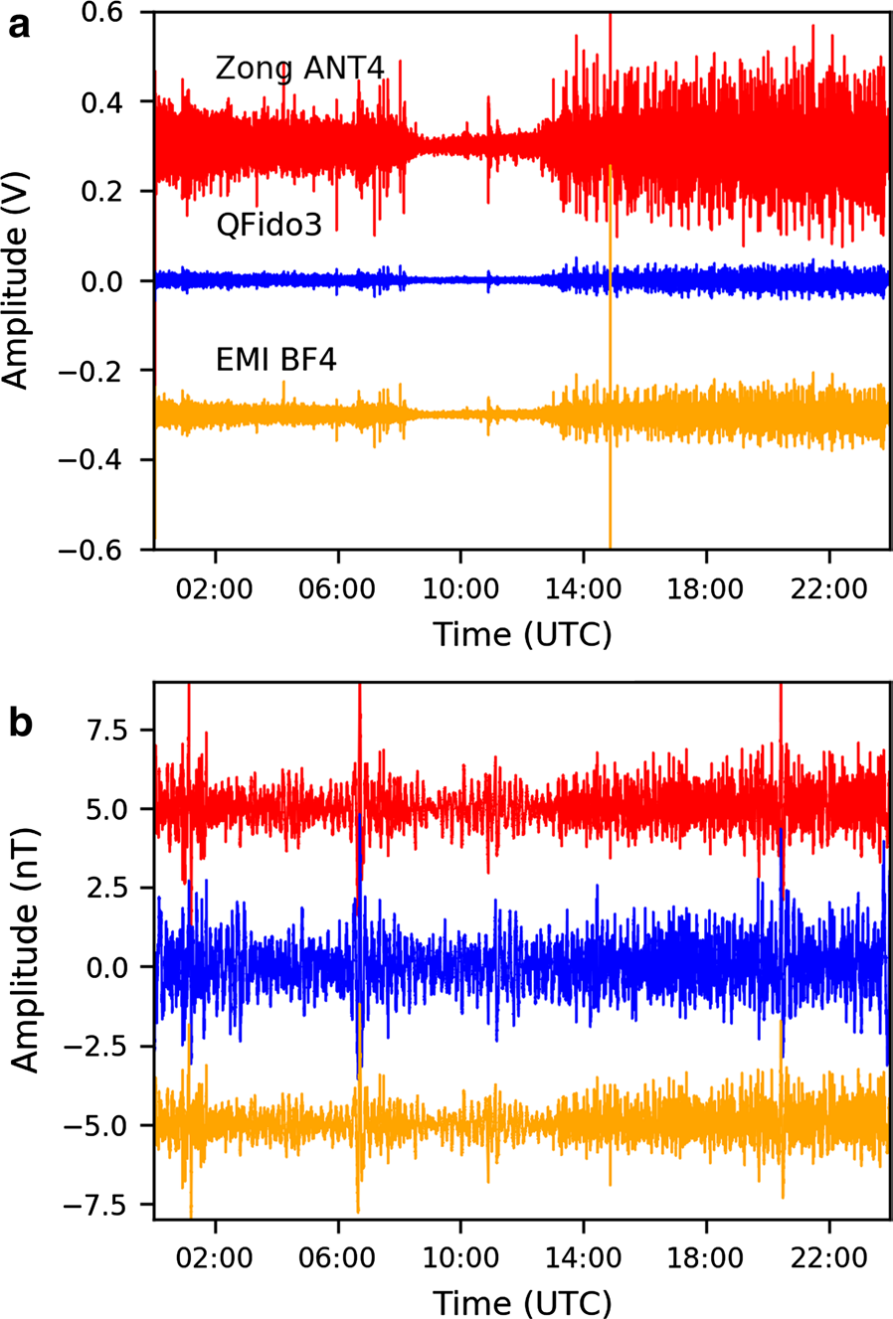
Day-long time series for April 12, 2014, recorded in **a** voltage and **b** nanotesla (i.e., after correction for instrument response). All data are from the north–south coils and are shown after a 1000-s high-pass filter. The time series are all zero-mean but are plotted with an offset for easier visual comparison

**Fig. 9:**
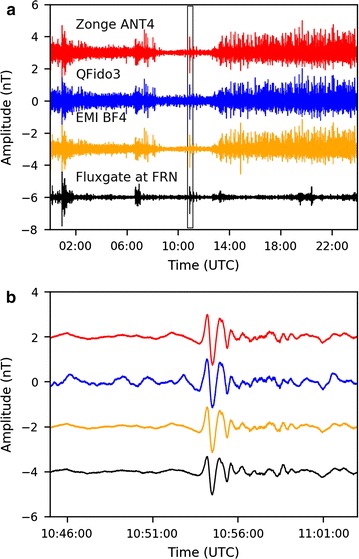
**a** Same time series as Fig. 8b but with a 200-s high-pass filter, and with the addition of the FRN fluxgate-magnetometer time series. **b** Expanded view of gray box in **a**, ~ 20 min of data, showing what we interpret as a Pi2 irregular geomagnetic pulsation spanning ~ 3 min

We compared north–south component magnetic field data recorded on all three coil types at JRSC with each other and with data from a remote reference site, FRN, ~ 225 km from JRSC (Fig. 1). Day-long voltage and nanotesla time series with a 1000-s high-pass filter (Fig. 8) demonstrated the importance of the instrumental transfer functions (Fig. 4): the night-time period that is evident as a quiet time in the voltage time series was enhanced in the nanotesla time series, in which the lowest decade of frequencies (0.001–0.01 Hz) had been boosted to its correct level (Fig. 6a), dominating the 0.01–0.1 Hz bandwidth of electric-train noise. When studying transient magnetic pulses, we commonly reviewed our time-domain data after a 200-s high-pass filter (Fig. 9a), allowing us to recognize anomalies, particularly during the quiet overnight period. One such significant disturbance during the quiet period (Fig. 8) could be clearly observed in the 200-s high-pass nanotesla time series and was also seen in the data from our remote reference site, FRN (Fig. 9a). Focusing on this disturbance (Fig. 9b), we found overall good coherence between FRN and all three sensors at JRSC in the 30–300 s period range, confirming that this is an external signal almost equally affecting all sensors across a region > 200 km. Based on the period, we identified this signal as a Pi2 irregular geomagnetic pulsation (e.g., Baumjohann and Nakamura 2009).

**Fig. 10:**
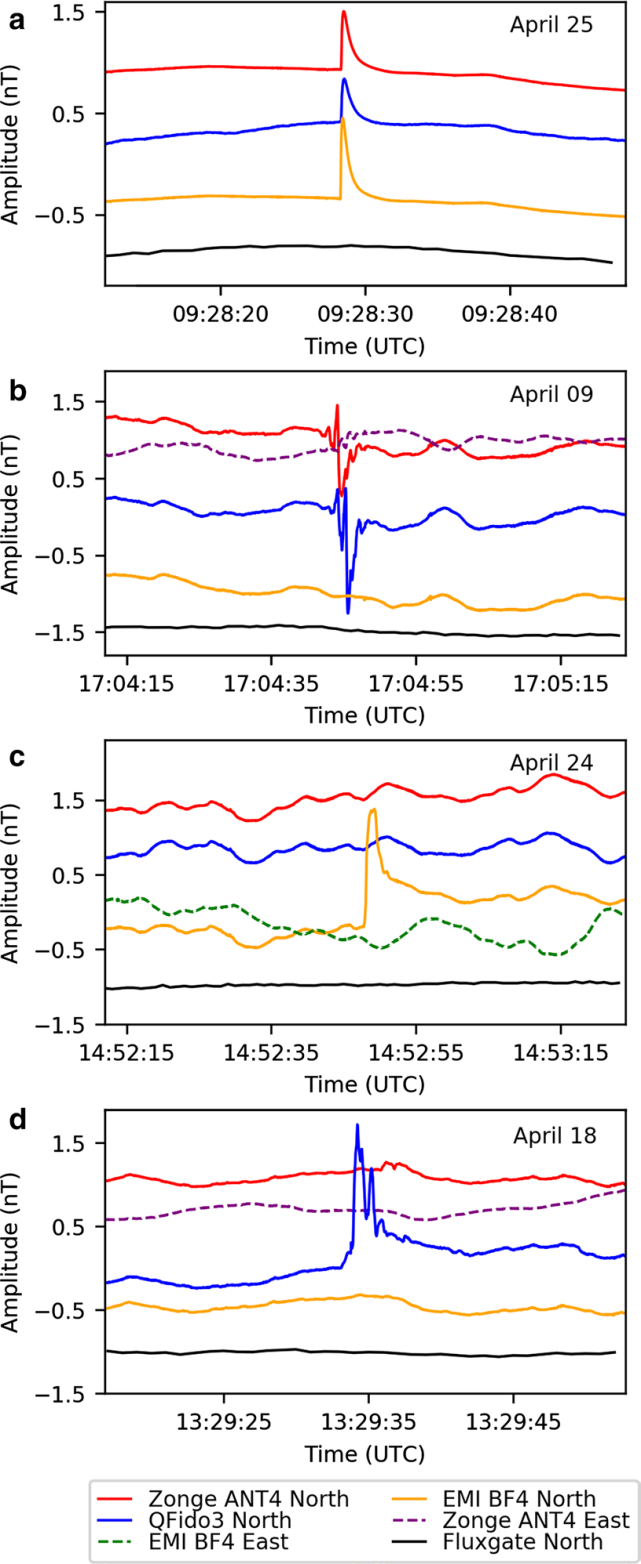
Comparison of individual pulses as recorded on different systems, plotted as in Fig. 9b, but for shorter time periods (pulses are 1–10 s in duration). **a** Single-side pulse recorded on all magnetometers at JRSC (Zonge ANT4, QFido3, EMI BF4) but not in Fresno, ~ 225 km distant. **b** Two-sided pulse recorded on all QuakeFinder sensors (Zonge ANT4, QFido3) but not on USGS-Stanford coils, ~ 50 m distant. **c** Pulse recorded only on USGS-Stanford north coil, but not on east coil ~ 5 m distant, nor on QuakeFinder sensors. **d** Signal seen on QuakeFinder QFido3 sensor, but not on Zonge ANT4 coil ~ 5 m distant, nor on USGS-Stanford EMI magnetometers. Traces from different sensors are all plotted at the same scale, but shown offset vertically for easier comparison. Where two components of the same instrument are shown (Zonge ANT4 N and E in **b** and **d**; EMI BF4 N and E in **c**) they are overlain without offset

**Fig. 11:**
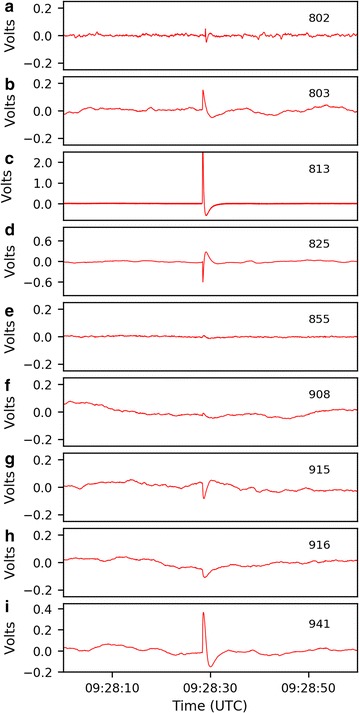
Time series recorded in voltage at nine nearby QuakeFinder stations. The pulse shown in Fig. 10a was detected at all nine QuakeFinder stations at 09:28:30 UTC, April 25, 2014. **a**–**i** Each station is labeled by station ID 802, 803, 813, 825, 855, 908, 915, 916, and 941, respectively

Not all signals observed at JRSC are atmospheric in origin, as attested by examples (Fig. 10) in which signals recorded at JRSC were not observed at remote reference FRN. Eight examples of similar signals were observed during a visual inspection of a 24 h segment of data, and six examples of such signals were observed when inspecting the quiet periods of each day (2 h, while BART is non-operational). These signals were absent from the FRN records but were recorded on all three separate sensor types at JRSC (Fig. 10a), demonstrating a local origin for these single-sided pulses. Although some signals with this shape (cf. Dunson et al. 2011) are known to be associated with lightning strikes (cf. Bleier et al. 2009), no strikes were recorded by a global lightning database (https://www.earthnetworks.com) within 500 km of the Bay Area corresponding with the time of this pulse. Instrumental noise could also be ruled out as the source, because the pulse was detected on all three coils and digitized on two separate systems. An assessment of records from nine nearby QuakeFinder stations (Fig. 11) revealed that the pulse was detected at all of the sites, but with varying amplitude and polarity. This indicates that the source was large enough to register on a regional-scale network; however, we could not determine whether this signal was cultural noise or a signal generated internally within the earth, without a more detailed analysis that would have been beyond the scope of this work. To make such a distinction would require a more careful cataloging of all nearby anthropogenic sources, as well as perhaps a temporal coincidence with a possible causative tectonic event. Alternatively, because the signal was recorded at a wide range of sites, the source of the signal could be deduced by inverse-modeling of current source shape and location (cf. Minamoto et al. 2011; Nagamachi et al. 2013). The sensor-spacing of such an array would need to be compatible with the nature of the sources. Although similar pulses have been suggested to have tectonic causes prior to earthquakes (Bleier et al. 2009; Dunson et al. 2011), in this case we suggest that a cultural noise source is a more likely origin. Irrespective of the cause of this pulse, the presence of the same signal on all three north–south sensors at JRSC is a positive demonstration that we can expect to successfully integrate data from different ULF networks despite their different equipment types.

In contrast to the desired—and expected—concurrence of the data from different sensors, in some instances signals were observed only on one system or one sensor, and were absent on the others, indicating either instrument noise (perhaps related to the power supply or digitizer) or extremely local phenomena (Fig. 10b–d). Figure 10b shows a complex 4-s signal occurring on the two QuakeFinder north–south sensors but not present (or highly attenuated) on the QuakeFinder east–west sensor, nor on any USGS-Stanford sensor. This two-sided pulse must have originated locally to the QuakeFinder instruments, since it was not visible 50 m distant on the USGS-Stanford sensors; moreover, it must also have been strongly directional and likely represented an electromagnetic source, since it was at least 20× larger on the north–south than on the east–west components. The time of day (09:04 local time) was consistent with an anthropogenic source. By contrast, a signal observed on the USGS-Stanford north–south sensor but not on any other sensors or systems (Fig. 10c), or on the QuakeFinder QFido3 but not on other sensors or systems (Fig. 10d), may represent system noise or perhaps physical disturbance to a single sensor (e.g., ground squirrel activity). Whereas the example in Fig. 10a is encouraging in demonstrating signal fidelity across independent systems, the examples in Fig. 10b–d are cautionary in demonstrating that there remain many individual signals that are clearly neither atmospheric nor tectonic in origin, which defy easy explanation.

## Data inter-comparison

The data described above (Fig. 10) imply that system noise or very local noise sources cannot be definitively excluded as the cause of any single anomalous pulse on a single sensor, or even of a pulse recorded on all sensors of a single magnetic station. Hence, attempts to find anomalies within long time series that could represent regional, even tectonic, sources must rely on comparison of different data streams. Perhaps the simplest method to explore the degree of similarity between different time series is a sliding-window cross-correlation, although of course arbitrarily more complex schemes can also be designed (cf. Kappler et al. 2017).

### Cross-correlations

**Fig. 12:**
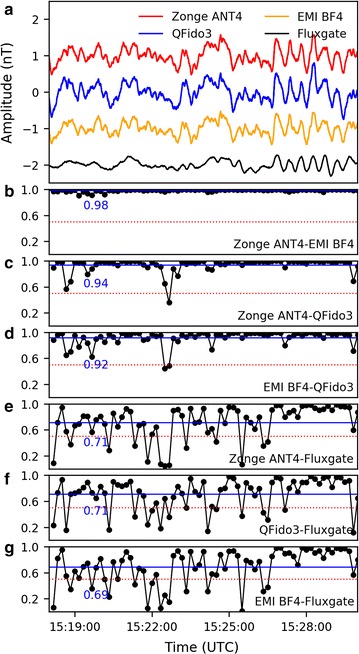
**a** Data for the period 15:18–15:30 UTC, April 17, 2017. **b**–**g** Cross-correlation coefficients calculated at 20-s intervals over the same time period between all pairs of north-oriented sensors. **b** Zonge ANT4 versus EMI BF4, **c** Zonge ANT4 versus QFido3, **d** EMI BF4 versus QFido3, **e** Zonge ANT4 versus Fresno Fluxgate, **f** QFido3 versus Fresno Fluxgate, **g** EMI BF4 versus Fresno Fluxgate. In each panel, the horizontal blue line shows and is labeled with the mean of the cross-correlation coefficients for the 12-min period, and the red dashed line shows an empirically estimated threshold of 0.5. Cross-correlations of USGS-Stanford and QuakeFinder records **b**–**d** are carried out with 200-s high-pass filtered data streams as used elsewhere in this paper; cross-correlations with the Fresno Fluxgate magnetometer that only records at 1 Hz sample rate **e**–**g** use a data stream bandpass-filtered with a 1–200-s Butterworth filter

We calculated the cross-correlation coefficients between all different pairs of sensors for random data segments to understand the typical degree of similarity, using 20 s windows with 10 s overlap on data high-pass filtered above 200 s. We additionally calculated the average correlation coefficient for longer time windows of interest. For a typical 12-min window (Fig. 12), in which we might hope for perfect correlation between stations separated ~ 50 m, we found a maximum average correlation coefficient of 0.98 between our two quietest coils (Zonge ANT4/EMI BF4) (Fig. 12b), and only slightly lower average values for cross-correlations of these two sensors with QFido3 (0.94 and 0.92; Fig. 12c, d). However, these slightly lower average correlation values masked several shorter intervals when the different sensor signals were not well correlated (e.g., values below 0.5 around 15:22:30; Fig. 12c, d), likely representing anomalous energy on the QFido3 sensor. In order to identify individual pulses of possible interest for earthquake prediction (Bleier et al. 2009), we would need to re-examine these data streams using correlation windows significantly shorter than the 20-s windows used here.

In order to extend this analysis to our remote reference station FRN, which was only sampled at 1 s, we band-passed the JRSC datasets from 200 to 1 s (Fig. 12e–g). As expected, average correlations between JRSC and FRN (~ 0.7, Fig. 12e–g) were lower than those between JRSC sensors (Fig. 12b–d), corresponding to the relative power of regional external signals and local anthropogenic signals. Individual cross-correlation values ranged from 0.99 to 0.05, suggesting that observing strategies comparing cross-correlations between near and far stations may be useful in detecting anomalous tectonic pulses, if they exist.

**Fig. 13:**
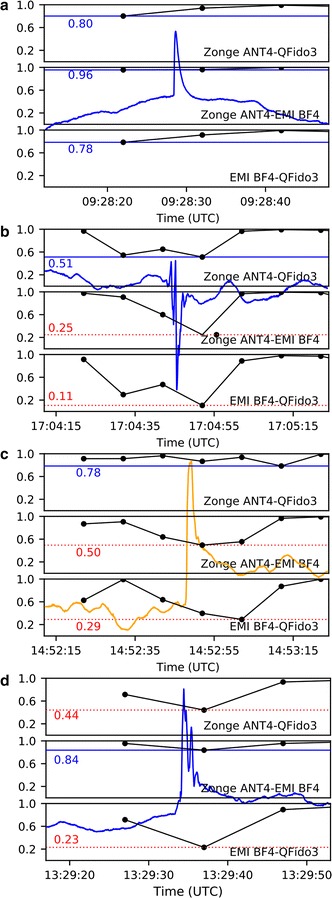
Cross-correlation coefficients calculated as in Fig. 12 for the data shown in Fig. 10, labeled **a**–**d**, as in Fig. 10. Horizontal lines show the minimum correlation coefficient in each data segment. The horizontal line is shown in solid blue when the minimum coefficient is greater than 0.5; otherwise, the horizontal line is shown as dotted and colored red. Background trace in **a**, **b**, and **d** is the QFido3 record (blue here and Fig. 10a, b, d); background trace in c is the EMI BF4 record (orange here and in Fig. 10c)

We next calculated cross-correlations for the four short time periods previously defined (Fig. 10) as showing specific local or system noise pulses (Fig. 13). Instead of calculating mean cross-correlation values, we identified the minimum cross-correlation values associated with the presence of spurious pulses observed on one or a few sensors. For the pulse observed on all three north–south sensors at JRSC (Fig. 10a), correlation coefficients between sensors were greater than 0.99 (Fig. 13a) when the pulse was active, only dropping to ~ 0.8 before or after the pulse. By contrast, cross-correlation coefficients never exceeded 0.5 for sensor pairs in which one trace had a spurious pulse (Fig. 13b–d), whereas in the case of sensor pairs nominally recording the same data, and lacking spurious pulses, minimum cross-correlations coefficients never dropped below 0.5. We suggest that thresholding the cross-correlations between pairs of stations, with the threshold value set empirically depending on station separation, may be a useful step in distinguishing system/cultural noise from internal (possible tectonic) signals.

### Variations in standard deviations of amplitudes

**Fig. 14:**
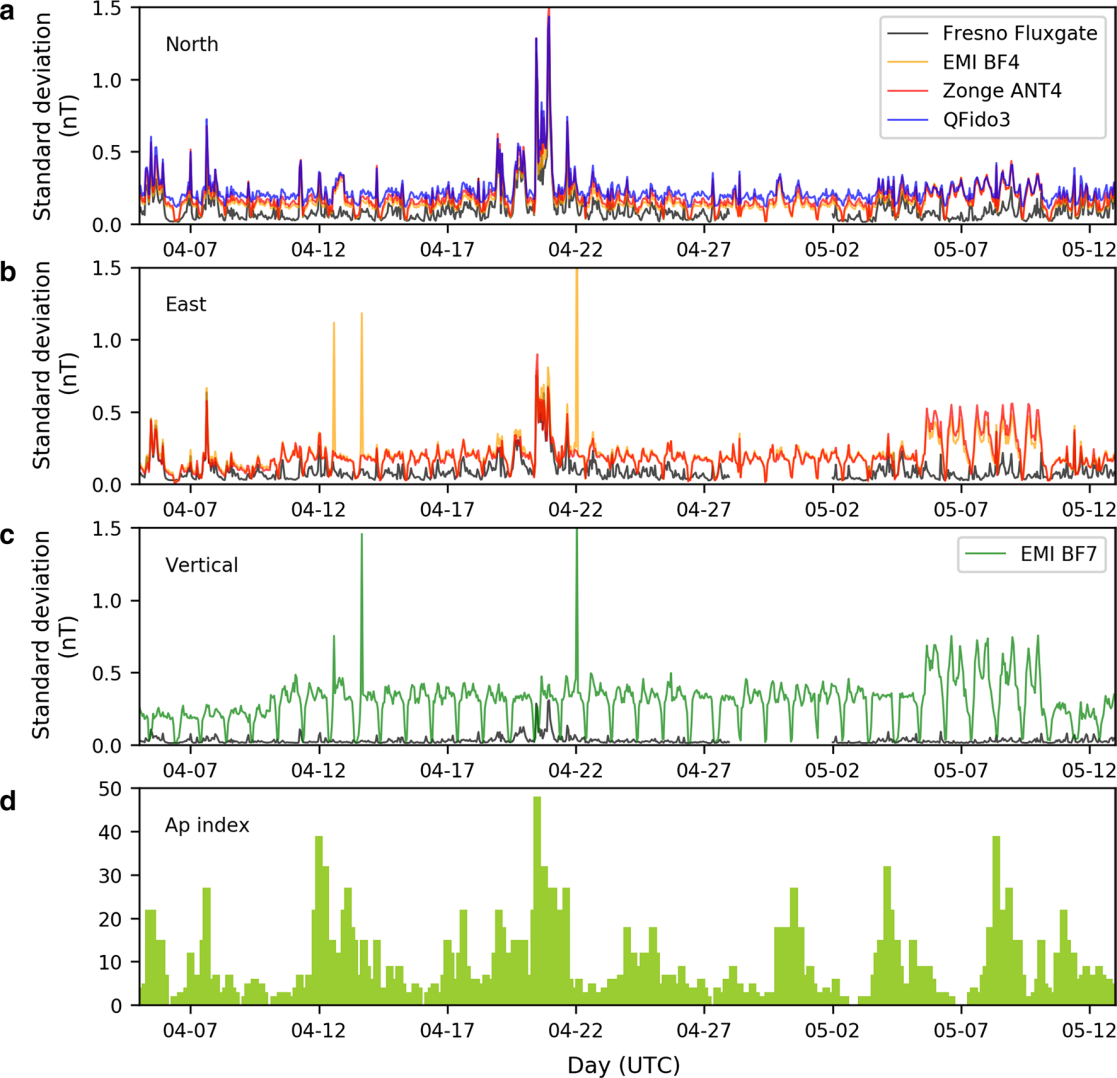
Standard deviations of amplitudes for each hour of data calculated individually for each sensor: **a** north-oriented magnetometers; **b** east-oriented sensors; and **c** vertical sensors, at Jasper Ridge and Fresno, for April 5, 2014 to May 12, 2014. Fresno data are not available for April 29, 2014 to May 1, 2014. **d** Geomagnetic activity index Ap for the same time period

In addition to carrying out running cross-correlations, we also estimated temporal signal stability on individual sensors over the entire 6-week deployment by calculating the standard deviation of signal amplitudes for each hour of data for each sensor. Amplitudes for all JRSC sensors, but not the FRN remote reference, were higher during the day than at night (Fig. 14a–c). As the increase in amplitude was contemporaneous, on a repeated daily basis, with the electric-train activity, we were confident that this amplitude increase represented anthropogenic noise.

We compared these time-varying standard deviations to the Ap (averaged planetary) index (Fig. 14d), a standard quantification of daily global geomagnetic activity as estimated from 13 geomagnetic observatories, varying from 0 to 400 (NOAA 2014). The National Oceanic and Atmospheric Administration defines days when Ap > 49 as major geomagnetic storms, and Ap > 29 as minor geomagnetic storms. We recorded the largest variance of all components of all systems on April 20, 2014, coinciding with the largest natural geomagnetic activity during our 6-week deployment (Ap = 48). A less pronounced correlation on April 12, 2014, showed a slight increase in the standard deviation during the second largest Ap index value (particularly on the east and vertical components). By contrast, a prominent 5-day-long variance increase at JRSC (May 6–May 10, 2014), which was not observed in the FRN data, strongly suggested a local and most likely anthropogenic source.

## Discussion and conclusions

Two independent ULF magnetic recording systems utilizing three different magnetic coil designs, representative of the QuakeFinder and USGS-Stanford networks, were collocated at Jasper Ridge Biological Preserve, Stanford University, in a successful attempt to characterize and cross-calibrate the different instruments. Although the systems showed remarkably similar time series (Figs. 9, 10a) and spectral responses (Figs. 6, 7), occasional signals in the time series occurred on only a single system or component (Fig. 10b–d), indicating sensor, system, or extremely local sources of noise. These pulses of energy emphasize the need for very thorough characterization of signals in order to understand their origin. Such familiarity with each system response to known signal sources will provide a well-defined context in which to distinguish potential future anomalies.

The overall data coherence between the three sensor types recorded on two digitizer models was sufficient to confirm that multiple independent stations and networks can and should be integrated in the search for tectonic magnetic signals. The multiple examples of unexplained pulses on just one or two of the six sensors deployed demonstrated the need for multiple scales of separation of different recording stations, in order to be able to separate individual sensor noise from multi-component system noise, local cultural noise, possible tectonic signals, and/or regional geomagnetic noise. Any successful identification of a tectonic magnetic signal should ideally include simultaneous detection of energy on more than one component of a multi-component system to isolate single-sensor noise (e.g., animal disturbance or amplifier problems) and on more than one station to exclude noise affecting an entire system (e.g., owing to power or digitizer problems), while failing to detect the same energy on a remote reference station to exclude natural geomagnetic variations. Thresholding running cross-correlations between pairs of parallel sensors at different stations/sites appears to be one simple way to rapidly assess data coherence and identify potential anomalies (Figs. 12, 13).

The distinction between local anthropogenic sources and local tectonic causes remains highly problematic and requires effort on two fronts. First, detailed analysis of long-term records at a single station should allow recognition of persistent anthropogenic signals (Fig. 7) and their exclusion from consideration as tectonic signals. Second, statistical analysis of any remaining potentially anomalous signals may be required, at least until theoretical developments can characterize an expected response time function for plausible tectonic scenarios.

Rather than hoping that the few existing and spatially dispersed magnetic recording stations will not only record but also identify anomalous tectonic signals, the community should increase its efforts to enhance and densify cross-calibrated recording systems in areas of likely future seismicity.

## Abbreviations

BART: Bay Area Rapid Transit
cpv: counts per volt
DC: direct current
FRN: USGS Geomagnetic Observatory at Fresno, CA
JRSC: USGS-Stanford electromagnetic observatory at Jasper Ridge Biological Preserve, CA
LT: local time
NOAA: National Oceanic and Atmospheric Administration
SLAC: SLAC Linear Accelerator Center
ULF: ultra-low frequency
USGS: United States Geological Survey
UTC: universal time coordinated
VTA: Valley Transportation Authority (Santa Clara, CA)
